# Cementing technique for total knee arthroplasty in cadavers using a pastry bone cement

**DOI:** 10.1186/s13018-021-02436-z

**Published:** 2021-07-01

**Authors:** Hans Bösebeck, Anna-Maria Holl, Peter Ochsner, Manuel Groth, Kevin Stippich, Andrej M. Nowakowski, Christian Egloff, Sebastian Hoechel, Beat Göpfert, Sebastian Vogt

**Affiliations:** 1 Heraeus Medical GmbH, Philipp-Reiss-Strasse 8/13, 61273 Wehrheim, Germany; 2 Universitätsspital Basel, Orthopädie, Rüttigasse 7, 4402 Frenkendorf,, Switzerland; 3 Kantonsspital Baselland, 4101 Bruderholz, Switzerland; 4 Universitätsspital Basel, Orthopädie, Spitalstrasse 21, 4053 Basel, Switzerland; 5 University of Basel, Musculoskeletal Research, Pestalozzistrasse 20, 4056 Basel, Switzerland; 6 University of Basel, Department Biomedical Engineering, Gewerbestrasse 14, 4123 Allschwil, Switzerland

**Keywords:** Pastry cement, PMMA, Total knee arthroplasty, Cemented TKA, Bone cement

## Abstract

**Background:**

In cemented primary total knee arthroplasty (TKA), aseptic loosening remains a major cause for failure. Cementing techniques and characteristics of a chosen cement play a key role for good fixation and implant survival. A pastry bone cement was developed to facilitate the cement preparation and to rule out most of preparation-associated application errors. The pastry bone cement was compared to a conventional polymethyl methacrylate cement in a TKA setting.

**Methods:**

Standardized implantations of total knee endoprostheses were performed in bilateral knee cadavers to investigate handling properties, variables of cement application, working time, and temperature development. Mechanical aspects and cementation quality were assessed by pull-out trials and microscopic interface analysis.

**Results:**

Both cements expressed similar characteristics during preparation and application, only the curing time of the pastry cement was about 3 min longer and the temperature peak was lower. Fractures of the conventional cement specimens differed from the pastry cement specimens in the tibial part, while no differences were found in the femoral part. Penetration depth of the pastry cement was similar (tibia) or deeper (femur) compared to the conventional cement.

**Conclusions:**

The pastry cement facilitates the feasibility of cemented TKA. The pre-clinical tests indicate that the pastry bone cement fulfills the requirements for bone cement in the field of knee arthroplasty. A clinical trial is needed to further investigate the approach and ensure patient safety.

**Supplementary Information:**

The online version contains supplementary material available at 10.1186/s13018-021-02436-z.

## Background

In the field of joint replacement surgery, cemented and cement-less (“press-fit”) techniques are used for implant fixation [1]. Even though the use of uncemented implants has increased in recent years, bone cement remains the predominant technique used in total knee arthroplasty (TKA). Aseptic loosening is the most frequent reason for TKA revisions [2, 3].

While some studies show that cemented TKAs have lower failure rates and greater functional outcomes compared to uncemented TKAs [4–6], others show similar outcomes for cemented and uncemented TKAs [7–13].

The widespread use of cemented TKA is supported by extensive clinical experience and provides the advantage of local antibiotic protection, if required. Advancements in cementing are usually referred to as first-, second-, or third-generation techniques. The developments include vast improvements in bone bed preparation, cement preparation, and cement delivery [14].

Although the underlying mechanism of aseptic loosening after TKA is not fully understood, it is commonly accepted that it has a multifactorial etiology [15] that can be patient and/or treatment related. In cemented TKA, better outcomes have been achieved with several technical improvements of the later generation techniques, including vacuum mixing, compression with a cement gun, cement precooling, and high-pressure lavage. The quality of the chosen cement also plays an essential role in prevention of aseptic loosening, as cement fractures and debonding between cement and implant are likely to initiate failure.

For the optimal outcome, knowledge about the bone cement is of paramount importance and a precise, standardized cementing technique is essential [14]. Various types of polymethyl methacrylate (PMMA) bone cements are commercially available, usually provided as two (liquid/powder) sterile components along with multiple additives. Dependent on the PMMA cement composition, usage and cement properties vary. PMMA cements express distinct characteristics regarding viscosity, curing process, temperature development, handling characteristics, cement structure, mechanical properties, and release capacity. However, even slight changes in the mixing ratio can affect the characteristics of a given cement tremendously. Despite several improvements during the recent years, cement preparation remains a source for mistakes.

In contrast to the conventional cement consisting of a powder and a fluid component, the pastry bone cement [16] is prepared by mixing two different paste-like components offering a ready to use cement option without the need of a mixing procedure, thereby facilitating cement preparation and limiting preparation-associated application errors.

The objective of this pre-clinical investigation was to evaluate the quality of cementation, handling and product properties, as well as the mechanical characteristics of this pastry bone cement compared to a conventional cement in a TKA setting.

## Materials and methods

### Investigation design

This pre-clinical investigation was conducted in the Anatomical Institute of the University of Basel, which furnished 16 medically prepared knees of 8 deceased persons without obvious pathological findings. Pre-trials were conducted on artificial and cadaver material in the sense of feasibility.

Standardized implantations of total knee endoprostheses with a bone cement paste and an established bone cement were performed in bilateral knee cadavers (prepared isolated cadaver material). On one side, the prostheses components were implanted with the conventional powder/liquid cement, while on the opposite side, the bone cement paste was applied under otherwise equal conditions. The selection was performed randomly and alternating.

### Medical devices

A pasty two-component bone cement [16] and the established cement Palacos® R+G (Heraeus Medical GmbH, Wehrheim), based on the powder-liquid system, were used in this pre-clinical investigation. Both devices are high-viscous polymethylmethacrylate cements with comparable component compositions and the same amount of gentamicin.

The implants used for the tibial and femoral parts were Duracon® (Stryker) and porous-coated anatomic (PCA) prosthesis (Howmedica), both with an identical porous coating. Either sample implants, or implants obtained by explantation during revision surgery were used. The components were purified thoroughly, adherent cement remnants were removed by incubation in acetone and mechanical post-processing, and final sterilization was achieved by gamma radiation.

### Preparation of cement

For both knee implants, 60 g of conventional cement (40 g powder + 20 g fluid) or of pastry cement (30 g paste A + 30 g paste B) were initially mixed according to the manufacturer specifications. The mixing procedure was performed by using the Palamix® (Heraeus Medical) System (hand mixing) without a vacuum. Cement application was executed by a conventional cement gun.

### Surgical approach

Investigation was executed on prepared isolated cadaver material (knee joints) that was fixed with a buffered formaldehyde solution. Detailed instructions were given during an onsite training performed by specialists from Heraeus Medical. Tendons, ligaments, and cartilage structures of the knee joint were removed. Bone was prepared without patella.

The surgical handling of the experiments was executed by two fully trained orthopedic surgeons specialized in TKA. Preparation of the bone surfaces: the valgus angle of the femur was 5°, femur size was measured, and the final preparation of the femur with the bevel saw cuts took place. Dorsal condyles were sawed off. The required tibial resection template was applied; a correct slope and the physiological tibial axis to the second (2°) toe were aligned. The tibial plateau was sectioned, the size was measured, and the prosthesis chosen. To remove loose cancellous bone, blood, fat, and marrow, all bone surfaces were washed with pulsatile lavage using warm tap water. Washing time (3 min) and water temperature (40–43 °C) were monitored.

**Fig. 1:**
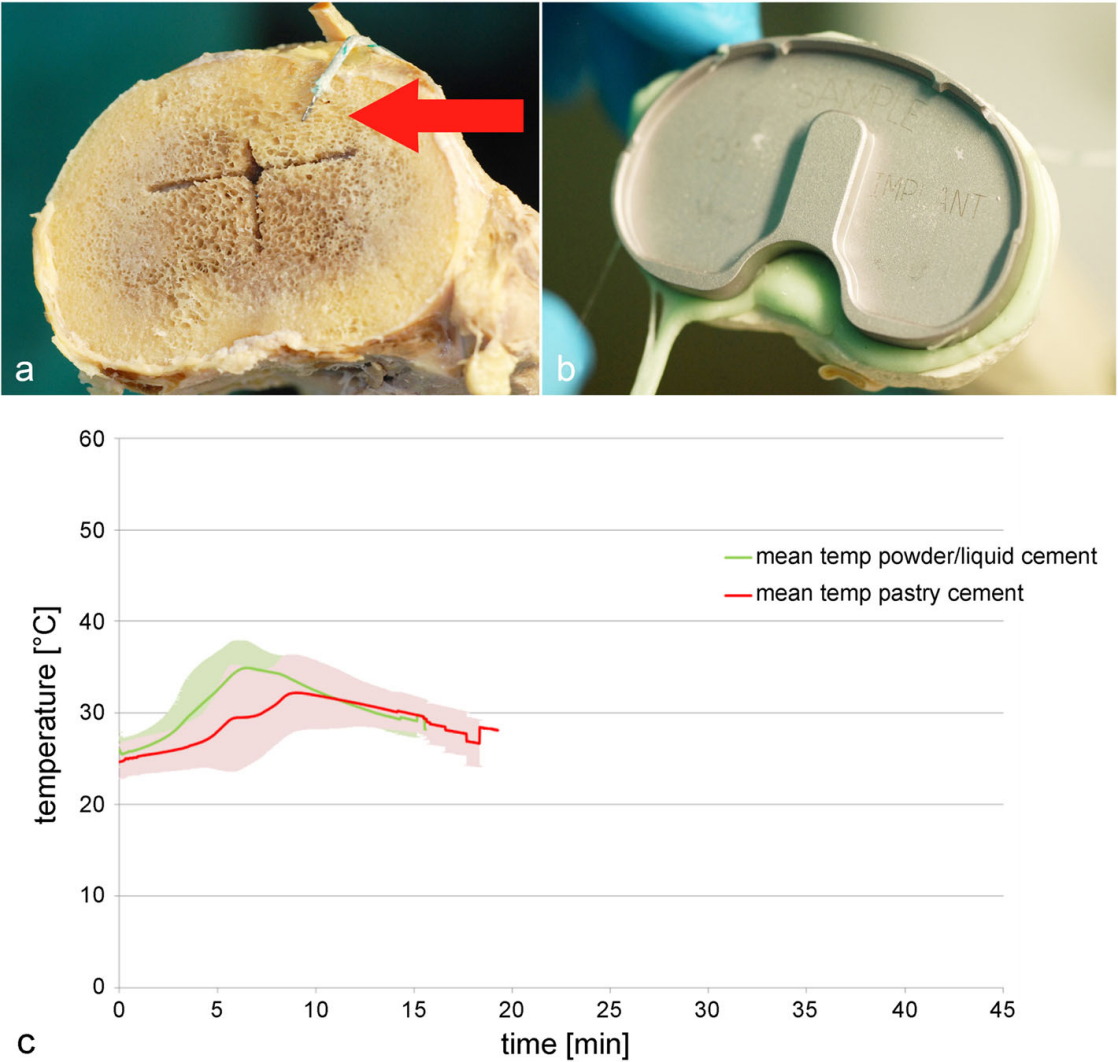
Tibial component. Location of thermo-sensor (**a**, red arrow), cement layer with implant (**b**), and curing temperature of cement (**c**). *N* = 8

The correct handling of the cement preparation was taken over by the Heraeus team. The tibial component was cemented first. Cement was applied early during its working phase onto the bone-side surface of the metal implant (sparing the cross entering in the tibial head), not on the bone surface (Fig. 1). As soon as the cement was no longer adhesive, it was manually shaped, matching the implant mold. The implant was inserted down into the hole for the tibial cross and onto the plateau. Holding pressure was applied and maintained until the cement had hardened to mimic the real-life situation. Cement curing was determined by a penetration test with a dissection needle and by the “ball-test” [17].

**Fig. 2:**
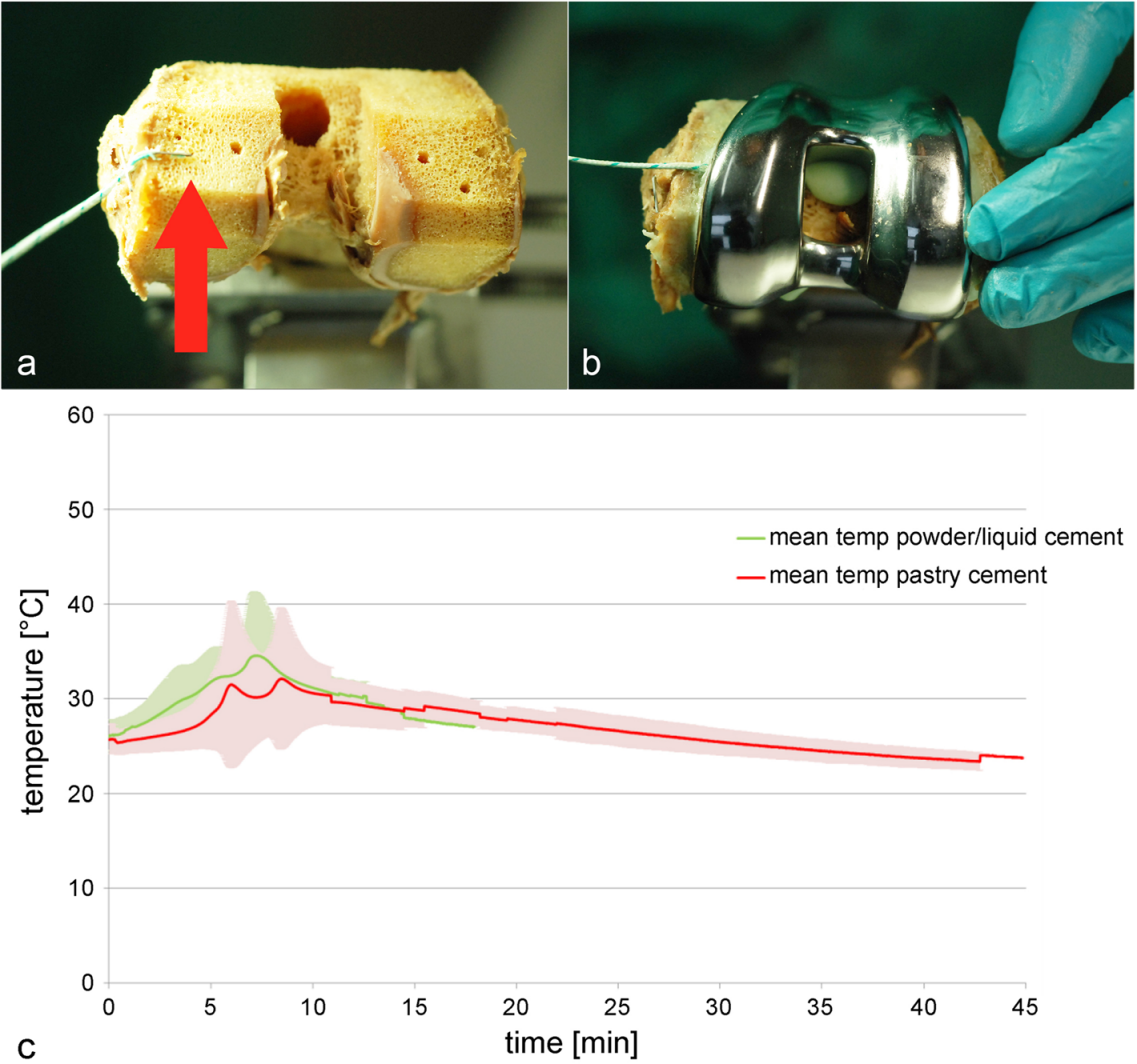
Femoral component. Location of thermo-sensor (**a**, red arrow), cement layer with implant (**b**), and curing temperature of cement (**c**). *N* = 8

The femoral component was cemented separately, but the procedure was analogous to the tibial component (Fig. 2).

To determine the total amount of used cement, the surplus of cement was measured, as well as all equipment in contact with cement before and after use, and subtracted from the initial amount.

### Overall assessment

Variables of cement application and working time were investigated. Maximum temperature was measured at the bone-cement interface with an implanted thermo-sensor (Testo-AG, 735-2) that was fixed by a clamp to keep it in place during the cementation process. The thermo-sensor was fixed at the location with the thickest layer of cement, where the highest curing temperatures were to be expected (Figs. 1 and 2). Bone cement mechanical properties, attributes, amount of applied cement, technical data, and user acceptance were tested.

### Pull out trials

Trials were conducted in the Center of Biomechanics, University of Basel. A servo-hydraulic testing machine (Typ MTS Bionix 858) was used to measure the maximum tractive force of 5 knee implants for each cement. Femur and tibia were fixed on a pipe socket by bolting and by applying a two-component adhesive based on epoxy resin (Sikadur-31 CF Normal, Sika AG, Zürich). Remaining space between bone and pipe socket was filled with the adhesive. According to the manufacturer, a tensile strength of 17–23 N/mm^2^ is achieved after a setting time of 3 days at room temperature.

To test the tibial component, the tibia-plateau was linked to the connecting element of the femur condyle. Similarly, the femur condyle was used with the corresponding connecting element for the femoral component trials. To allow proper fixation, plastic shims were inserted into little notches of the prostheses medially and laterally of the tibia-plateau and femur condyle during cementation. They were removed after curing of the cement, to allow the positioning of the pincers.

Surgical tools (Stryker) were used as joints to connect the implants with the testing machine. The tractive force and the displacement speed of the axial cylinder were recorded at sampling rate of 1000 Hz, and the whole process was documented with high-speed filming (300 frames per second). After fixation on the testing machine, human preparations were exposed to tractive forces of 20 N. Subsequently, tractive forces were increased constantly (1 m/min) until implant-cement-bone anchoring failed.

### Microscopic interface analysis

One cadaver was used for analysis (male, 69 years). Pastry cement was applied at the left side, and conventional powder/liquid cement at the right side.

All preparations were sectioned with a diamond tipped bend saw. To prevent melting of the cement, cutting was water-cooled and slow (about 12 h per sample). The femoral longitudinal cut was frontal through the anchorage pins. The tibial longitudinal cut was frontal, right behind the frontal metal anchor. All segments were analyzed with a Keyence microscope.

### Statistical methods

Only descriptive statistical methods were used in the present pre-clinical investigation. Continuous parameters are presented with their means, standard deviations (SDs), medians, and minimum and maximum values. Frequencies were calculated for categorical variables.

## Results

### Factual investigation

Sixteen knee joints from eight prepared cadavers were used for the evaluation of TKA. The demographic data are shown in Table 1. One cadaver was female, seven were male.

**Table 1:** Demographic data

Variable	*N*	Mean (SD)	Median	Min; Max
Age [years]	8	76.8 (13.19)	82.0	57; 93
Body size [cm]	8	172.9 (7.41)	174.5	162; 183
Body weight [kg]	8	71.0 (10.61)	68.0	59; 85
BMI [kg/m^2^]	8	23.81 (3.55)	25.35	17.9; 26.8

*BMI* body mass index, *Max* maximum, *Min* minimum, *N* number of non-missing values, *SD* standard deviation

The mean valgus angle was 8.1 ± 1.57° for the pastry cement and 6.8 ± 3.33° for the conventional cement. Mean values for the length of caput femoris–condyles medialis femoralis and condyles medialis tibiae–pilon tibiale are shown in Table 2. Mean width of the femoral condyles and the tibial plateau were about 81 mm for both treatment groups. In the OP room, mean temperature was 20.8 °C for both cements. The mean humidity was 65 ± 1.31% for the pastry, and 64.3 ± 1.28% for the conventional cement.

**Table 2:** Length and diameter of the leg

Variable	Medical device	*N*	Mean (SD)	Median	Min; Max
Caput femoris–condylus medialis femoralis [mm]	Pastry cement	8	473.1 (± 27.33)	466	437; 513
	Powder/liquid cement	8	474.4 (± 22.35)	469	448; 505
Femoral condyles (width) [mm]	Pastry cement	8	80.74 (± 5.102)	80.1	73.8; 89.2
	Powder/liquid cement	8	81.29 (± 5.107)	81.9	73.0; 87.0
Condylus medialis tibiae–pilon tibeale [mm]	Pastry cement	8	372.0 (± 28.46)	361	345; 414
	Powder/liquid cement	8	367.4 (± 28.13)	356	339; 404
Tibia plateau (width) [mm]	Pastry cement	8	80.60 (± 3.954)	79.6	75.6; 88.3
	Powder/liquid cement	8	81.56 (± 4.842)	81.3	74.2; 90.2

### Surgical preparation

For both cement types, the mainly used implant for the tibial part was Duracon® (Stryker) (six implants [75%] each). The other used implant was the PCA prosthesis (Howmedica) (two implants [25%] each). In case of the pastry cement, Duracon® was used during all surgical preparations of the femoral part (eight implants [100%]), while Duracon® (seven implants [87.5%]) and PCA (one implant [12.5%]) were used for the conventional cement.

The mean preparation time of the tibial condyles was about 5.5 min (pastry cement: 5.7 ± 2.91 min; powder/liquid cement: 5.45 ± 1.79 min), and about twice as long for the femoral condyles (pastry cement: 10.05 ± 3.06 min; powder/liquid cement: 13.06 ± 6.09 min). In all cases, mean lavage washing time was 3 min, and mean temperature was approximately 43.5 °C, except for the pastry cement during the preparation of the tibial part (40.4 ± 8.25 °C). The mean preparation time for the device ready to use was shorter for the powder/liquid cement compared to the pastry cement for both tibial (1.07 ± 0.12 min; 1.96 ± 1.36 min) and femoral condyles (1.02 ± 0.06 min; 1.81 ± 0.95 min). The mean weight of the application system plus cement (ready to apply) for both condyles was around 179 g for the powder/liquid cement and 188 g for the pastry cement. Immediate loss of stickiness after extrusion was documented for both cement types during all surgical preparations of the tibial and femoral part.

### Cement application—tibial part

A mean duration of cement application of 0.60 ± 0.17 min for the pastry cement and 0.62 ± 0.19 min for the powder/liquid cement was revealed. The mean time to implant set for the pastry cement was 1.77 ± 0.37 min and 1.48 ± 0.71 min for the powder/liquid cement. A longer time from start of the application to curing (trocar test) of about 3 min was shown for the pastry cement (mean value: 10.46 ± 2.01 min) compared to the powder/liquid cement (mean value: 7.53 ± 0.37 min). The mean really applied amount of cement during the surgical preparations of the tibial part was 9.06 ± 1.43 g for the pastry cement and 11.04 ± 1.55 g for the powder/liquid cement.

For both cement types, the time to place the metal implant was sufficient during all surgical preparations of the tibial part. The mean maximum temperature before the cementation process was 24.78 ± 1.79 °C for the pastry, and 25.49 ± 1.39 °C for the powder/liquid cement. Mean temperature peaks of 33.90 ± 4.15°C (9 min) and 36.28 ± 1.91 °C (6.5 min) were measured for the pastry and powder/liquid cement, respectively. The mean maximum temperature after the cementation process was 29.10 ± 1.93 °C for the pastry cement and for the powder/liquid cement it was 29.24 ± 1.28 °C (Fig. 1).

### Cement application—femoral part

The mean duration of cement application was 0.61 ± 0.21 min for the pastry cement and 0.7 ± 0.27 min for the powder/liquid cement. Mean time to implant set was 1.8 ± 0.69 min for the pastry cement and 1.83 ± 0.47 min for the powder/liquid cement. Comparable to the tibial part, a longer time from start of the application to curing (trocar test) of about 3 min was shown for the pastry cement (mean value: 10.21 ± 0.94 min) than for the powder/liquid cement (mean value: 7.47 ± 0.88 min). The mean really applied amount of cement during the surgical preparations of the femoral part was 10.96 ± 2.35 g for the pastry cement and 12.41 ± 1.46 g for the powder/liquid cement.

For both cement types, the time to place the metal implant was sufficient during all surgical preparations of the tibial part. The mean maximum temperature before the cementation process was 25.71 ± 1.55 °C for the pastry, and 26.16 ± 1.64 °C for the powder/liquid cement. Mean temperature peaks of 35.93 ± 9.16 °C (8.5 min) and 36.93 ± 5.91 °C (7.5 min) were measured for the pastry and powder/liquid cement, respectively. The mean maximum temperature after the cementation process was 27.39 ± 3.88 °C for the pastry cement and for the powder/liquid cement it was 29.51 ± 1.61°C (Fig. 2).

Thus, the total amount of either pastry of powder/liquid cement used was about 20 to 23 g per knee, which is roughly 30% of the initially prepared 60 g of cement. For all surgical preparations, no evaluation of the amount of cement needed for facultative Patella remodeling was performed.

### Properties of cement

For the pastry cement, the cement mantle was free of blisters at 6 implanted cadaver sides. For 2 implanted cadaver sides, concerning data were missing. An excellent adhesiveness to the implant was revealed at all 8 implanted cadaver sides.

For the powder/liquid cement, the mantle was free of blisters at all 8 implanted cadaver sides. The adhesiveness was mainly fair (5 implanted cadaver sides [62.5%]), followed by excellent (2 implanted cadaver sides [25%]), and poor (1 implanted cadaver side [12.5%]).

For both cement types, it was easy to remove the surplus cement and the prosthesis was assessed to be in position at all implanted cadaver sides. It was easy to manually transfer and mold the cement from gloves onto the implant surface. Only non-latex gloves (Biogel®) were used during the implantations.

### Overall assessment

The stress level for the user was low and the handling of the cement was easy at all implanted cadaver sides for both cement types.

A higher percentage of unproblematic methyl methacrylate (MMA) odor was revealed for the pastry cement (87.5%) compared to the predicate device (62.5%). The MMA odor was assessed to be tolerable for both cement types at the remaining implanted cadaver sides.

### Pull-out trial

**Fig. 3:**
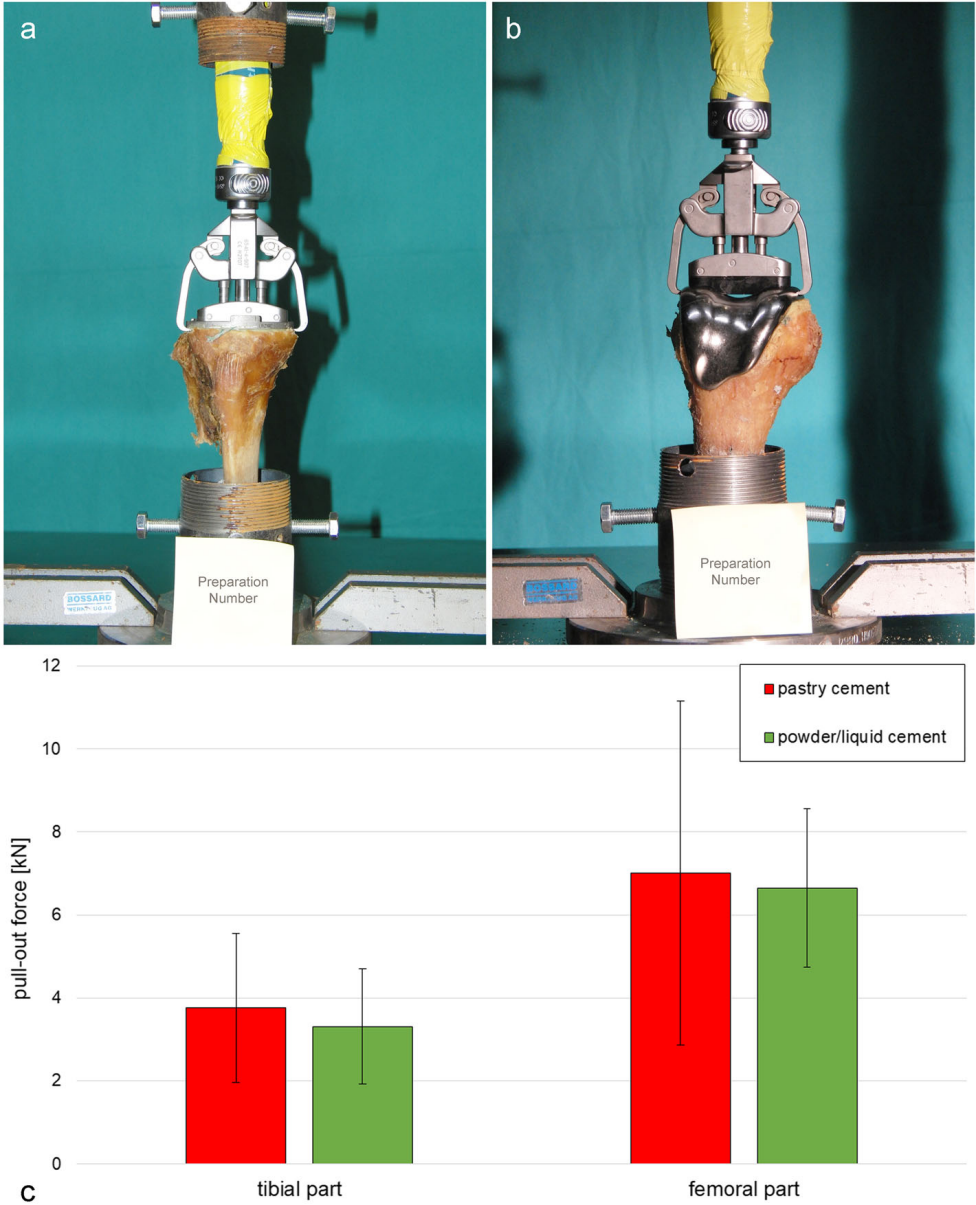
Tibia (**a**) and femur (**b**) fixed on the pipe socket and mean pull-out forces (**c**). *N* = 5. (the value of the femoral part from cadaver no. 82 [powder/liquid cement] was not considered due to failure of the implant).

In the tibial part (Fig. 3a), fractures of the powder/liquid cement specimens (Suppl. 1) were located mostly along the implant-cement-bone interface. Part of the fracture took place between implant and cement and continued often into the bone-cement interface at the central, cross-formed shaft. For the pastry cement (Suppl. 2), fractures mainly occurred in the bone-cement interface. The mean pull-out force was slightly higher for the pastry cement (3764 ± 671 N) compared to the conventional powder/liquid cement (3312 884 N) (Fig. 3c).

In the femoral part (Fig. 3b), the fracture surfaces of the powder/liquid (Suppl. 3) and pastry (Suppl. 4) cement specimens were mostly across the condyle. The drill holes for fixation were often still visible. In one exceptional case (pastry cement), the fracture went through the implant-cement interface. The mean pull-out force was 7008 ± 2552 N for the pastry cement and 5964 ± 3364 N for the powder/liquid cement (Fig. 3c).

### Microscopic interface analysis

**Fig. 4:**
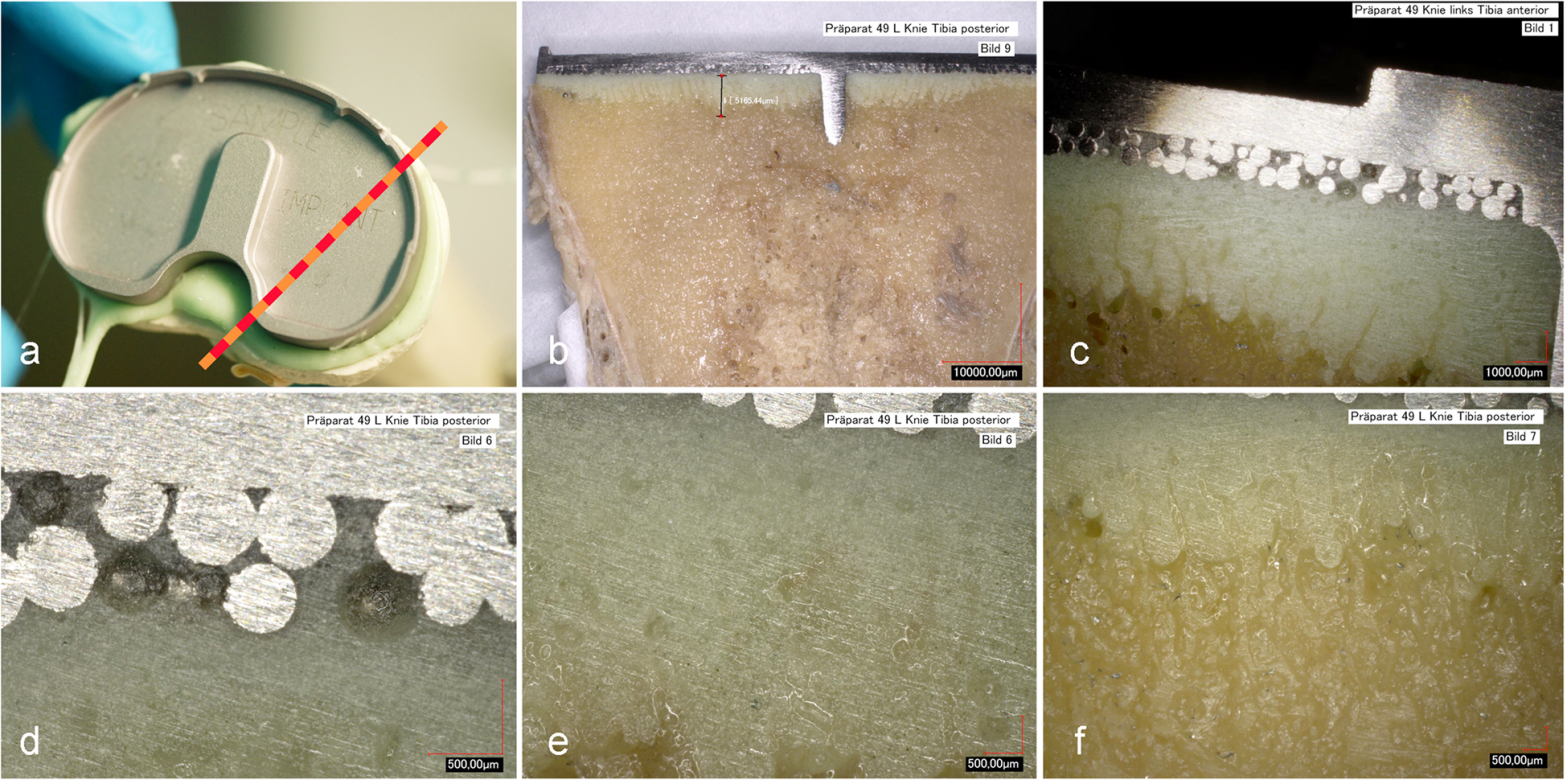
Interface analysis of the tibial part. Red bar marks the cutting line (**a**). Overview of the cut surface (**b**). Close-up of implant-cement-bone interface (**c**). Close-up of implant-cement (**d**), cement (**e**), and cement-bone interface (**f**). Figures are representative for powder/liquid and pastry cement

The cut surface of the tibia had a slightly concave shape (Fig. 4). Both cement types accumulated in the center of the plateau and the cement was thicker compared to the periphery. In the center, penetration depth was about 3 mm, and about 1 mm at the lateral and medial side. Similarly, penetration was fully achieved at the spheroidal structure of the bottom side of the implant, while it remained incomplete at the periphery. A small gap was detected between bone and cement on the edge of the implant.

**Fig. 5:**
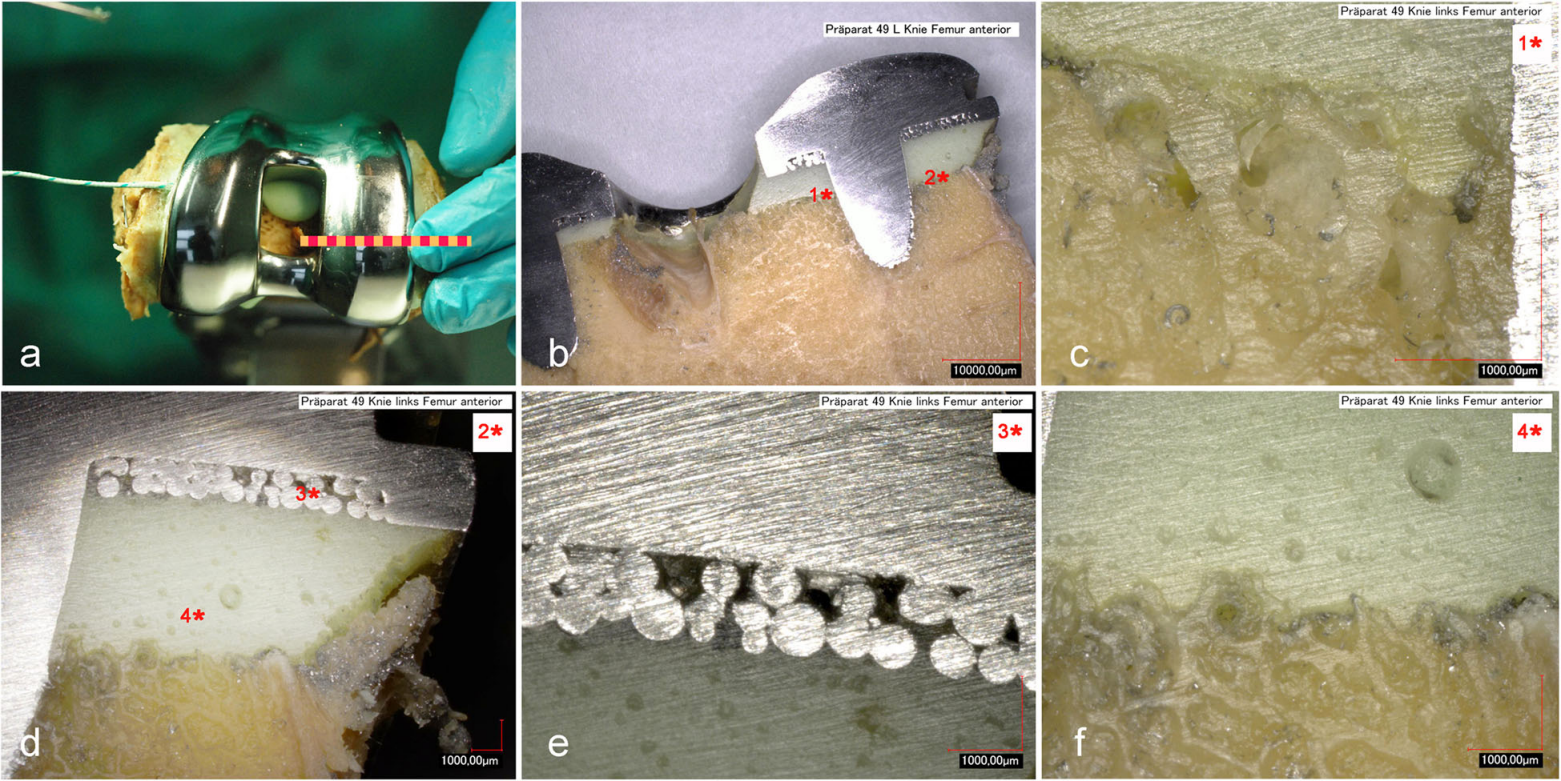
Interface analysis of the femoral part. Red bar marks the cutting line (**a**). Overview of the cut surface (**b**). Close-up of area 1* (**c**) and 2* (**d**) from the cut surface (**b**). Close-up of implant-cement interface area 3* (**e**) and cement bone interface area 4* (**f**) from figure 9d. Figures are representative for powder/liquid and pastry cement

In the femoral part (Fig. 5), penetration depth of the powder/liquid cement differed along the bone, with 0.67 mm medially and 0.47 mm laterally. Penetration depth of the pastry cement was 1.36 mm medially and 0.39 mm laterally. Accordingly, the cement thickness was bigger laterally then medially for both cements.

## Discussion

In the present study, a pastry bone cement was introduced and compared to a conventional PMMA cement, aiming to refine cemented TKA. Regarding the cementing technique, bone preparation and surgical approach, initial conditions were simulated as closely as possible.

Both cements expressed similar characteristics during preparation and application, only the curing time of the pastry cement was about 3 min longer for the tibial and femoral parts. The prolonged curing time may increase cement flow, thereby facilitating penetration. However, this was not confirmed by the present results.

The temperature was measured where cement was the thickest and heat development produced by PMMA curing was expected to be maximal. Temperature peaks were about 34 to 36 °C for the pastry cement and about 36 to 37 °C for the powder/liquid cement, the peaks were rather short-lived. In an animal model, it was shown that bone formation is reduced after 1-min exposure to a temperature between 47 and 50 °C, while no effects were observed at 44 °C [18]. Thermal bone necrosis starts only at 55 °C after 30 s of exposure [19]. The extent of thermal damage is both temperature and time dependent. The present results suggest that the risk of thermal injuries is low, which is in line with previous findings [20], but the thermal injury safety margin is narrow. It must be emphasized that the measured temperature is different from the real-life situation, since the initial cadaver temperature varied about 10 °C from the temperature of patients. Under physiologic conditions, vascularization will buffer the heat, but the cooling effect may be lower in the knee joint in case of an inflated tourniquet.

In earlier pull-out trials, it was shown that less dense bones with wide cancellous clefts allowed large cement pegs extending into the cancellous bone, entailing interface fractures that predominantly occurred within the bone. In dense bones, fractures occurred rather in the cement [21, 22]. In this study, fractures of the powder/liquid cement specimens differed from the pastry cement specimens in the tibial part, while no differences were found in the femoral part. Although bone density was not investigated directly, the devices were implanted in a random and alternating manner, ensuring comparable conditions. The results indicate that the cement-implant interface may be stronger for the pastry cement, but this can only be described as a tendency.

PMMA is used to tightly fill the space between the irregular bone surface and the implant. The penetration depth of cement into bone is supposed to be crucial for increased implant stability [23, 24]. Penetration depths of 2 to 4 mm into the proximal tibia is regarded as suitable for optimal fixation [25, 26], while penetration beyond 5 mm may increase the risk of thermal damage [27]. In addition, the degree of bone cement interdigitation may further affect the tensile strength of the cement-bone interface [28]: Microscopic interface analysis revealed that although cement was only applied onto the implant, both cements achieved a penetration depth of 3 mm in the center of the tibial part. Laterally, penetration depth was about 1 mm. In the femoral part, penetration depth of the pastry cement was centrally twice as deep as the powder/liquid cement. Again, penetration depth was lower in the periphery, suggesting that applied pressure was stronger centrally than laterally. However, patient-related conditions (e.g., bone density) and differences in the treatment (e.g., pulse lavage, tourniquet, surface drilling, use of laparotomy sponges, and suction) will lead to different penetration depths of the cement in vivo [29].

Pull-out trials and microscopic interface analysis indicate that the cement-bone/cement implant contact area is important for the interfacial strength as well. The findings are in line with Waanders et al., showing that cement penetration depth as well as contact area are key elements for optimizing the interfacial strength [30].

This study has several limitations. Obviously, the knee is exposed to a variety of motions, shear forces, and tensile or compressive loadings that cannot be assessed by pull-out trials. Moreover, the present study does not consider biological reactions due to polymerization heat, trauma, or monomer toxicity and clinical tests are needed to address these issues.

## Conclusion

The pre-clinical tests reported here show equal or even slightly improved properties of the pastry cement compared to the powder/liquid cement, indicating that the pastry bone cement fulfills the requirements for bone cement in the field of knee arthroplasty. As an elaborate mixing procedure (e.g., vacuum pump) is not needed for the pastry cement and the operator needs to perform only a few simple steps, the potential risk of cement-related failures is reduced. A clinical trial is needed to further verify the system.

## Supplementary Information

**Additional file 1.** Tibial part; powder/liquid cement. Pull-out forces of single specimens and correlating surface pictures after fractures. *Pipe socket moved slightly.

**Additional file 2.** Tibial part; pastry cement. Pull-out forces of single specimens and correlating surface pictures after fractures. *Pipe socket moved slightly.

**Additional file 3.** Femoral part; powder/liquid cement. Pull-out forces of single specimens and correlating surface pictures after fractures. *Pipe socket moved slightly; **failure of the implant (82L, notch for pull-out clamp broke).

**Additional file 4.** Femoral part; pastry cement. Pull-out forces of single specimens and correlating surface pictures after fractures. *Pipe socket moved slightly.

## Data Availability

Not applicable.

## Abbreviations

MMA: Methyl methacrylate
PCA: Porous coated anatomic
PMMA: Polymethyl methacrylate
SD: Standard deviation
TKA: Total knee arthroplasty
