# Supplementary figures and images for: Cementing technique for total knee arthroplasty in cadavers using a pastry bone cement
Source: J Orthop Surg Res. 2021 Jul 1;16:417. doi: 10.1186/s13018-021-02436-z (PMC8247244; doi:10.1186/s13018-021-02436-z)

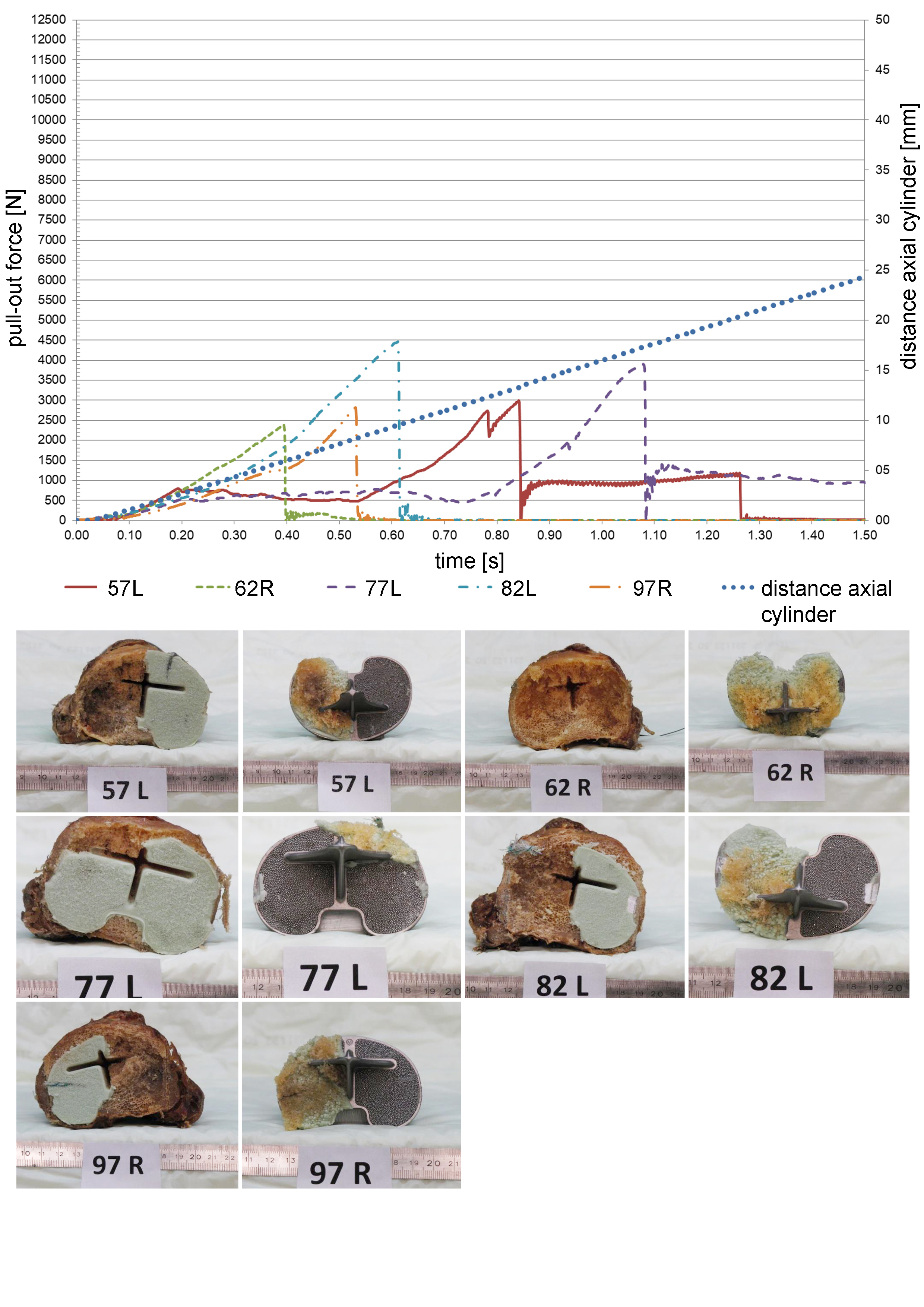

Supplement: Supplementary file 1 — Additional file 1. Tibial part; powder/liquid cement. Pull-out forces of single specimens and correlating surface pictures after fractures. *Pipe socket moved slightly. [file 13018_2021_2436_MOESM1_ESM.jpg]

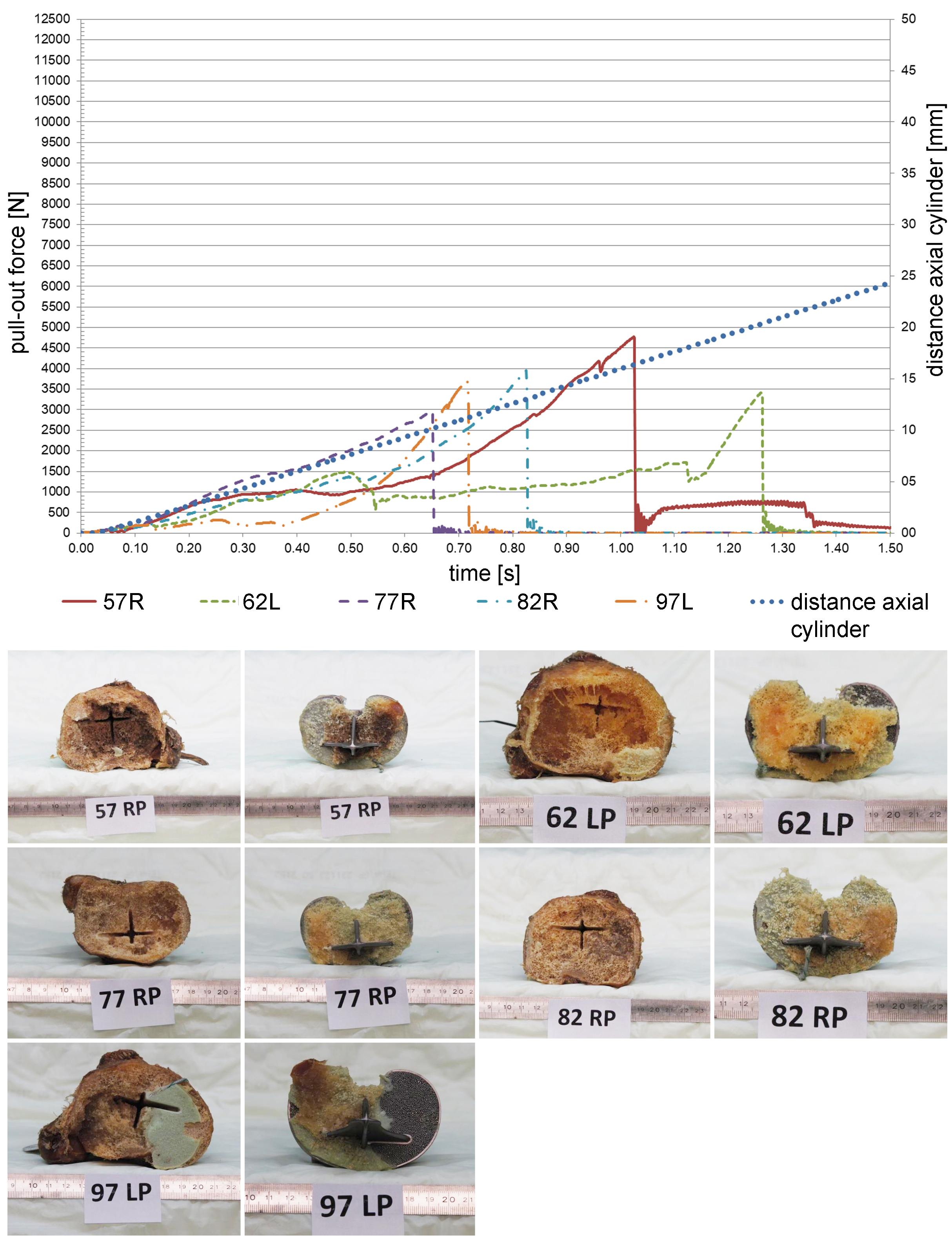

Supplement: Supplementary file 2 — Additional file 2. Tibial part; pastry cement. Pull-out forces of single specimens and correlating surface pictures after fractures. *Pipe socket moved slightly. [file 13018_2021_2436_MOESM2_ESM.jpg]

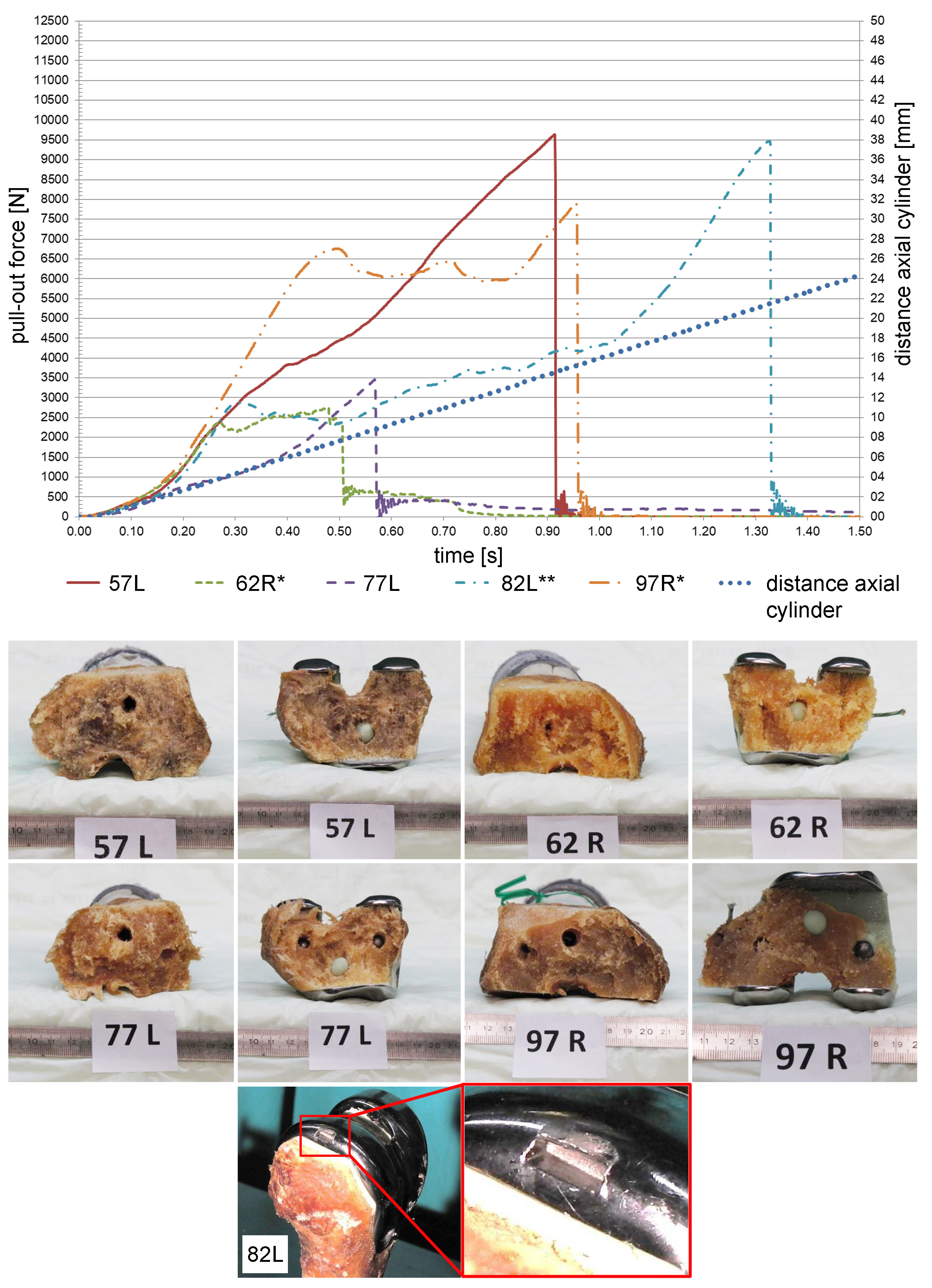

Supplement: Supplementary file 3 — Additional file 3. Femoral part; powder/liquid cement. Pull-out forces of single specimens and correlating surface pictures after fractures. *Pipe socket moved slightly; **failure of the implant (82L, notch for pull-out clamp broke). [file 13018_2021_2436_MOESM3_ESM.jpg]

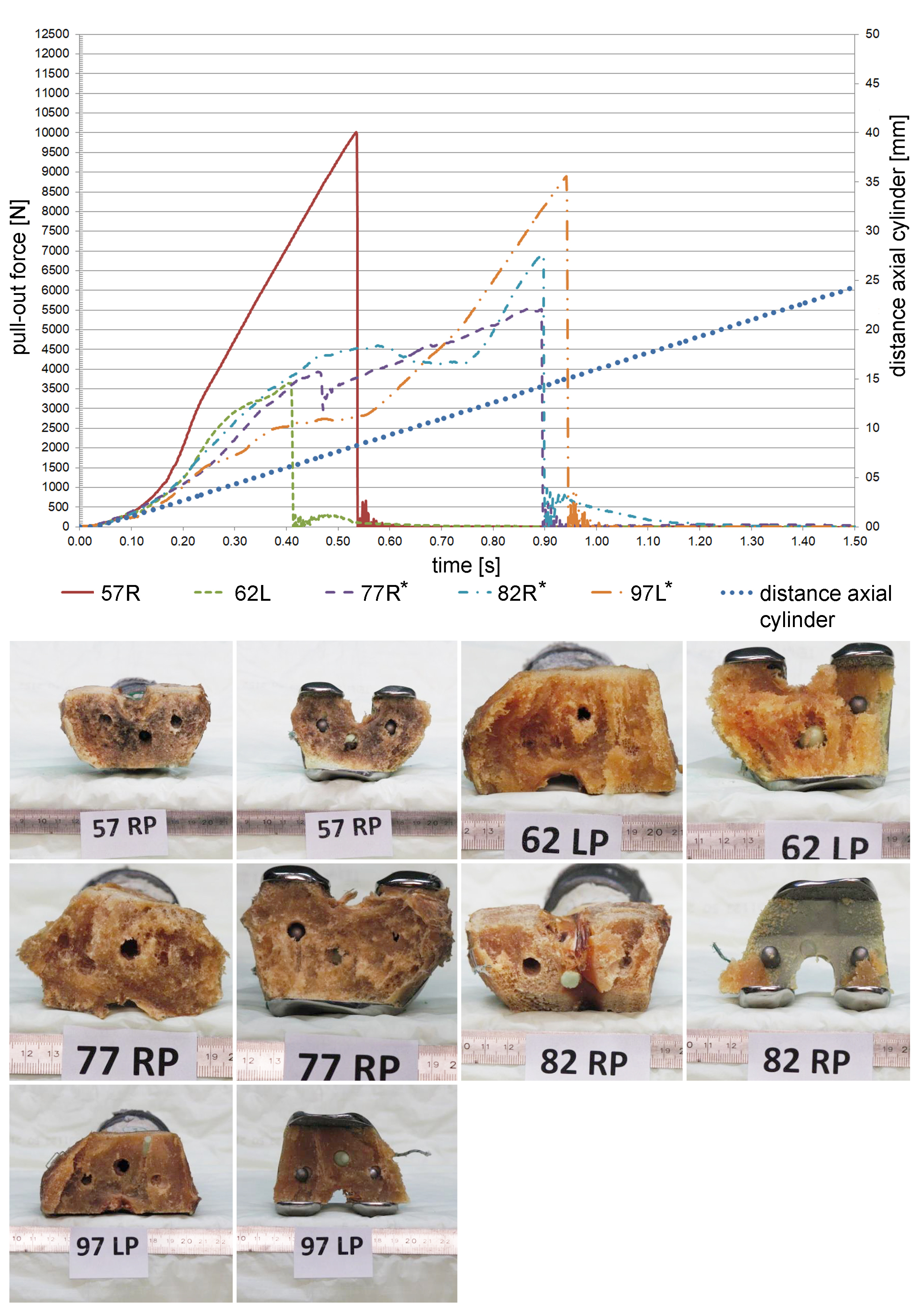

Supplement: Supplementary file 4 — Additional file 4. Femoral part; pastry cement. Pull-out forces of single specimens and correlating surface pictures after fractures. *Pipe socket moved slightly. [file 13018_2021_2436_MOESM4_ESM.jpg]
